# Lockdown impact on lifestyle and its association with oral parafunctional habits and bruxism in a Spanish adolescent population

**DOI:** 10.1111/ipd.12843

**Published:** 2021-06-18

**Authors:** María Carrillo‐Diaz, Ana Raquel Ortega‐Martínez, Martín Romero‐Maroto, María José González‐Olmo

**Affiliations:** ^1^ Orthodontic and Pediatric dentistry Department Rey Juan Carlos University Alcorcón Spain; ^2^ Psychology Department Jaén University Jaén Spain

**Keywords:** anxiety, bruxism, confinement, COVID‐19, internet use, social networking

## Abstract

**Aim:**

To analyse the possible association between decreased physical and social activity and an increase in the use of mobile devices, internet, and social networks with increased anxiety and the appearance of oral parafunctions and bruxism for adolescents before and during COVID‐19.

**Design:**

A total of 213 adolescents attended private clinics at two different times: before lockdown (T0) and after completion of total lockdown (T1). In T0 and T1, a clinical examination was carried out to assess dental wear (IA) and only in T1 were they given the self‐report questionnaire which focuses on the periods before and during lockdown (self‐reported bruxism, state anxiety, mobile phone and internet, social network use, physical and social activity, and questions on parafunctions).

**Results:**

There were a decrease in physical activity at T0‐T1 and an increase in social media use, internet, state anxiety, and clinical and self‐reported bruxism at T0‐T1. There was a positive correlation between increased self‐reported bruxism, increased social media use, mobile device use, and state anxiety.

**Conclusions:**

There has been a change in adolescent lifestyle during lockdown with an increase in the prevalence of oral parafunctions and bruxism. In particular, the increase in the use of social networks at night and also in anxiety levels during lockdown were associated with of the increase in self‐reported bruxism.

**Why this paper is important to paediatric dentists**

- There has been a change in adolescent lifestyle during COVID‐19 lockdowns in terms of limited physical activity, poor social life, increased mobile phone use, decreased social networks, and internet abuse. There has been an increase in the occurrence of oral parafunctions and bruxism during lockdown.
- The increase in the use of social networks at night and the increase in anxiety during lockdown were associated with increase in self‐reported bruxism by the patient.
- Additionally, the increased use of social networks at night plays a moderating role between the increase in state anxiety and the increase in bruxism.

## 1 INTRODUCTION

Due to the rapid spread of the SARS‐CoV‐2 virus, responsible for the COVID‐19 pandemic, Spanish authorities imposed strict regulatory measures aimed at preventing the transmission of the virus. One of these measures was an obligatory total lockdown from 16 March to 4 May.

The interruption to education and physical activity in the home lockdown of children and adolescents has led to a decrease in socialization and to an increase in uncertainty and anxiety.^1^

In this scenario, technology enables adolescents to contact and communicate with others via internet and telephone, which in fact is considered helpful as a means of stress reduction.^2^ A problem arises, however, when use becomes compulsive.

Recent studies warn of the risks that occur when adolescents become consumed by their online activity, one of which is developing symptoms of pathological internet use (PIU),^3^ also described in the literature as problematic use of the internet.

The same applies to smartphones. Their abuse among the adolescent population has become one of the major concerns of the last decade, and in particular, the possible connection that overuse of telephones and a sedentary lifestyle have had with adverse pathologies such as physical health and emotional problems, anxiety,^4^ sleep disorders and academic failures.^2^, ^3^, ^4^

Being confined leads inevitably to a sedentary lifestyle and the likely adoption of undesirable habits such as the excessive use of screens.^5^ In addition, physical activity is essential for psychological and physiological health^6^(maintenance of muscular functions, bone health, body composition, etc) and may also have an impact on the quality of sleep.^7^

Thus, an odontological question arises : Is there a risk of developing or aggravating bruxism and/or certain oral parafunctions in the adolescent population as a result of lifestyle changes associated with the pandemic?

Parafunctional habits are a multifactorial problem whose aetiology is not yet fully understood. Most theories that have attempted to explain their appearance suggest several factors to be involved: odontological factors (skeletal malocclusions or occlusal alterations),^8^ psychosocial factors (such as depression, anxiety, and stress),^9^ and sleep‐related^10^ factors, which are thought to be precursors of bruxism.

Therefore, given that parafunctions and bruxism affect the adolescent population and that some of the possible factors involved (psychosocial and sleep disorders) have possibly increased as a result of lifestyle change during the lockdown, this study has been proposed with the following objectives:

- To assess whether there has been a change in the lifestyle of adolescents during lockdown in terms of decreased physical activity, poor social life, cell phone, social network, and internet abuse.
- To establish whether there has been a greater occurrence of oral parafunctions and bruxism during lockdown.
- To analyse the possible association between decreased physical activity and social life, an increase in the use of mobile devices, internet, and social networks with increased anxiety, and the appearance of oral parafunctions and bruxism.
- To determine what lifestyle changes after lockdown are able to predict the appearance of bruxism.
- To ascertain whether the increased use of social networks at night is a moderating factor between the increase in both state anxiety and bruxism.

## 2 MATERIAL AND METHODS

Ethical approval was obtained from the Research Ethics Committee of the Universidad Rey Juan Carlos, Spain. Participants were informed about the objectives and nature of the study and were assured of the confidentiality of the information collected. Finally, all participants signed an informed consent form before their inclusion in this study. The parents or guardians who agreed to participate signed an informed consent form (under 14 years old), and the adolescents were also asked to give their own consent (14 years or older).

Recruitment of participants was carried out by selecting all those adolescents who had attended appointments between September and December 2019 (T0) at private clinics in Madrid. An incidental non‐probabilistic sample of 213 participants of both genders was used. The data were collected by a single examiner. A repeated‐measures design was used with two time points: before lockdown (T0) and after completion of total lockdown (T1). Between T0 and T1, 12 months elapsed.

In both T0 and T1, a clinical examination was performed to determine dental wear. But only in T1 were they given the self‐reporting questionnaire which focuses on the periods before and during lockdown.

The inclusion criteria were the following: willingness of the minor to participate, parental consent for the minor's participation in the study, age between 11 and 17 years, fluent Spanish speaker, and good general health. The exclusion criteria were the following: being in orthodontic treatment at the time of data collection, having some dental pain, having temporomandibular dysfunction, ASA>I, presence of systemic disorders (cardiovascular, pulmonary, neuromuscular, and digestive), and/or mental development, neurological, and/or neuropathic pain, being under medication that alters the neuromuscular system, which could interfere with the central nervous system.

A clinical examination was performed at the clinic, in which the index of clinical bruxism was recorded. One of the team's researchers recorded the total number of teeth and the severity of enamel erosion on each tooth: level 0 (no obvious enamel wear), level 1 (enamel to dentin wear at single points), level 2 (dentin wear down to a third height of the crown), and level 3 (more than a third of tooth's crown or restoration material worn).

The tooth wear index was calculated with the method proposed by Ekfeldt et al^11^ It is a reliable index, previously used in literature for the clinical diagnosis of bruxism, and displays a significant association with bruxism. It is determined as the index IA = (10 × G1 + 30 × G2 + 100 × G3)/(G0 + G1 + G2 + G3), where IA is the tooth wear index and G0, G1, G2, and G3 are the number of teeth with level scores 0, 1, 2, and 3, respectively. In the clinical examination, the presence of hypertrophy of the masticatory muscles was recorded as well as indentations in the tongue or lip and/or a dawn line on the inside of the cheek.

On completion of this, a self‐reported questionnaire was given consisting of five sections: (a) a Self‐reported Bruxism Questionnaire (SBQ); (b) questions about parafunctions; (c) State Anxiety Scale (STAI‐S); (d) use of day and night networks; (e) specific questionnaires on information and communication technologies (CERI and CERM); (f) Physical Activity Questionnaire (IPAQ‐SF); and (g) Social Participation Questionnaire (SSPQ).

A Self‐reported Bruxism Questionnaire (SBQ) was collected to assess participants' self‐perception of bruxism. The questionnaire consists of 11 items that group the most common questions for the self‐reported diagnosis of bruxism.^12^, ^13^, ^14^ Examples of which include the following: ‘*Have you noticed that you grind or clench your teeth frequently during sleep?’ ‘Has anyone heard you grind your teeth at night?’ ‘Has your jaw felt tired or painful when waking up in the morning?’* A 5‐point Likert response system is used graded on the intensity (1 = nothing to 5 = a lot). The score is obtained after adding up the 11 items. With a range of 11‐55 points, the SBQ presented a reliability coefficient of α = 0.88.

To evaluate the presence of parafunctions such as nail, lip, or the biting of hard objects, questions such as: ‘*Do you bite your tongue and/or lip?’ ‘Do you bite your nails?’ ‘Do you bite hard objects?’* were used with a dichotomous yes/no answer.

The Spanish version of the State Anxiety Questionnaire (STAI‐S) was used to measure anxiety state. The STAI‐S is a self‐report questionnaire composed of 20 items under the instruction ‘right now, at this moment’. It uses a 4‐point Likert‐type response system scaled according to the intensity (0= almost never/nothing, 1= somewhat/sometimes, 2= quite often, 3= very much/almost always). The total score ranges from 0 to 60 points.^15^

Studies carried out on the Spanish population show adequate levels of internal consistency and adequate psychometric indicators.^15^ The internal consistency of state anxiety for our sample was α = 0.85 and α = 0.82, respectively, for T0 and T1.

The patterns of use of ICT (information and communication technologies) were identified through the CERI (Questionnaire of Experiences Related to Internet Use) and CERM (Questionnaire of Experiences Related to Cell Phones).^16^ The CERI and CERM questionnaires contain 10 items on a 4‐point Likert scale (1: never/almost never, 2: occasionally, 3: sometimes, 4: almost always). An example of an item in the CERI is as follows: ‘*How often do you stop the things you are doing to stay connected to the network longer?’* An example of item in the CERM is as follows: ‘*Do you get angry or irritated when someone bothers you while using your mobile?’*

The result of the score is the sum of the answers of all the items. With a range of 10‐40 points, higher scores indicate higher levels of internet and telephone use. The reliability analysis obtained values α = 0.79 for CERI and α = 0.81 for CERM at T0 and T1.

Two measures previously used by Woods & Scott, 2016^17^ were collected to assess social media use. The term social networking refers to social networking sites (eg Facebook, Instagram, and Twitter) and instant messaging (eg WhatsApp, Snapchat, and Facebook messenger). One of the measurements was for overall social media use and the other for night‐time social media use.

The first measured overall social media use and consisted of 6 questions about frequency and duration of social media use. A 6‐point Likert scale from ‘Less than once a month’ to ‘Daily’ was used. With a range of 0‐30, a higher score indicates a higher use of social media in general. The second measured night‐specific social media use and consisted of 7 questions about frequency of social media use shortly before bedtime and in bed; duration of social media use after bedtime; perceived delays in sleep due to social media use; and frequency and duration of sleep disturbances due to social media alerts. A Likert scale of 6 points from ‘Never’ to ‘Daily’ was used. Each scale gave an overall score of 0‐35, with higher scores indicating higher levels of social media use. Cronbach's alphas were 0.78 and 0.76 on overall and evening social media use, respectively, at T0 and T1.

In addition, the level of physical activity was collected with the International Physical Activity Questionnaire in its short version (IPAF‐SF).^18^ The IPAQ‐SF is a self‐report questionnaire that assesses physical activity in the last 7 days. The validity of physical activity assessed with the IPAQ‐SF has been previously reported.^18^ The IPAQ‐SF records the number of days per week and the minutes per day devoted to physical activity in four degrees of intensity: sitting, walking, moderate intensity (eg leisure cycling), and vigorous intensity (eg running or aerobics). The energy expended was estimated in hours of metabolic energy equivalent (MET) per week in the following manner, METs walking =3.3 × (minutes walking) × (days walking in leisure time); METs moderate =4.0 × (minutes of moderate intensity activity) × (days of moderate intensity leisure); METs vigorous =8.0 × (minutes of vigorous intensity activity) × (days of vigorous intensity leisure). The total minutes of leisure METs per week was estimated as follows: total METs = (walking METs) + (moderate METs) + (vigorous METs).

The Social Participation Questionnaire (SSPQ) is a short 14‐item questionnaire modified from the original Social Participation Index.^19^ From questions 1 through 12, the participant could choose one of six response categories: ‘Never’=1 item; ‘Rarely’=2 items; ‘Sometimes’=3 items; ‘Often’=4 items; and ‘At all Times’=5 items. For the remaining two questions, a binary response of ‘Yes’=5 points/‘No’=1 point was requested. The total scores of this questionnaire correspond to the sum of the points obtained in the 14 questions. The total SSPQ scores range from ‘14’ to ‘70’, where ‘14’ indicates that the participant has ‘never’ been socially active, a score between ‘15’ and ‘28’ indicates that the participant has ‘rarely’ been socially active, a score between ‘29’ and ‘42’ indicates that the participant is ‘sometimes’ socially active, a score between ‘43’ and ‘56’ indicates that the participant is ‘often’ socially active, and a score between ‘57’ and ‘70’ indicates that the participant is ‘always’ socially active. Cronbach's alphas in our study were 0.88 and 0.81, respectively, for T0 and T1.

### 2.1 Statistical analysis

The study is based on a pre/post design, which considers the variables described in the previous section. A statistical analysis was performed using SPSS version 26 (SPSS Inc.). The data analysis included descriptive statistics and the Kolmogorov‐Smirnov test to evaluate the assumption of normality, which was confirmed. To ascertain possible differences in T0‐T1 for continuous variables, paired t tests were performed. According to Cohen (1988), small Cohen's d values are ≈0.2, medium values are ≈0.5, and high values are ≈0.8. Cohen (1988) also considers small effect size values to be ≈0.01, medium values to be ≈0.06, and those large enough to be taken into account as ≈0.14. Significance levels were established at 0.05. A regression analysis determined which factors are predictors of the increase in self‐reported bruxism.

Subsequently, a PROCESS module (version 3.3) by Hayes was used to perform multiple simple moderation analyses (model 1) using SPSS. This was done to observe whether the increased use of social networks at night is a moderating factor between the increased state anxiety and the increase in the Self‐reported Bruxism Scale.

## 3 RESULTS

The sample comprised 213 adolescents, 116 females and 97 males. The age range was 11‐17 years, with a mean of 14 (±1.9) years.

### 3.1 Lifestyle Change

As described in Table 1, there was a significant decrease in physical activity (t(212) = −22 096, *P* < .01) and social activity (t (212) = 51 881, *P* < .01) in T1 compared to T0, as well as a significant increase in the frequency of daytime social networking (t (212) = −14. 718, *P* < .01) and night‐time (t (−17 542)=, *P* < .01), of the use of internet (t (212) = −13 254, *P* < .01), of the mobile telephone devices (t (212) = −13,678, *P* < .01), and of state anxiety (t (−39 646)=, *P* < .01). Moderate/large effect sizes were observed in all comparisons.

**TABLE 1:** Comparison of the variables of physical activity, social activity, daytime social network use, night‐time social network use, CERM, CERI, Anxiety State, Bruxism Index, and Self‐reported Bruxism Scale before and after confinement

	T0 M (SD)	T1 M (SD)	*t*	*P*‐value for change (T0‐T1)	*D Cohen*
Physical activity	856.6 (343.5)	332.8 (91.6)	22.096	*P* < .01	2.08
Social activity	51.3 (6.9)	21.7 (4.1)	51.881	*P* < .01	5.21
Use of daytime social networks	14.2 (6.04)	18.1 (9.2)	−14.718	*P* < .01	0.50
Use of social networks at night	7.9 (8.3)	20.7 (9.9)	17.542	*P* < .01	1.40
CERM	18.4 (7)	22.1 (8.6)	−13.254	*P* < .01	0.47
CERI	23.6 (3.9)	26.8 (4.6)	−12.188	*P* < .01	0.75
Anxiety state	18 (10.3)	32.7 (13.4)	−39.646	*P* < .01	1.23
Bruxism index	0.5 (0.3)	0.8 (0.6)	−7.935	*P* < .01	0.63
Self‐reported Bruxism Scale	10.4 (7.3)	15.4 (13)	−6.188	*P* < .01	0.47

Note

*d* de Cohen = TE small ≈ 0,20; TE medium ≈ 0,50; TE large ≈ 0,80.

### 3.2 Oral parafunctions and bruxism

In addition, during lockdown the number of adolescents biting their nails increased by 11.3% (N = 24), also the number of those biting their tongue increased by 9.4% (N = 20).

When comparing the values obtained in T0 and T1, a statistically significant increase was found in the index of clinical bruxism (t (212) = −7,935, *P* < .01) and in the scale of self‐reported bruxism (t (212) = −6188, *P* < .01). Moderate effect sizes were observed in comparisons.

### 3.3 Lifestyle change and increased oral parafunctions and bruxism

To those participants who registered an increase in nail biting during lockdown, there was also a higher state anxiety (19.7 ± 7) in comparison with those who did not start the habit (14.1 ± 4.8) (t (211) = −4.975, *P* < .01). Those who started the habit of nail biting also had a higher use of daytime social networks (5.7 ± 3.5) than those who did not (2.7 ± 3.3) (t (211) = −4.206, *P* < .01). Large effect sizes were observed in the comparisons.

Pearson's product‐moment correlation was run to assess the relationship between the change in physical activity, social life, daily and nightly social network use, cell phone, and internet use and the increase in the clinical bruxism index and the Self‐reported Bruxism Scale. In addition, the relationship between the increase in state anxiety and the increase in the clinical bruxism index and the Self‐reported Bruxism Scale was assessed.

As described in Table 2, there was a moderate and statistically significant positive correlation between the increase in the Self‐reported Bruxism Scale, the increase in the use of day and night social networks, the CERM, and state anxiety.

**TABLE 2:** Intercorrelations between Λ T0‐T1 variables studied (physical activity, social activity, daytime social networking use, night‐time social networking use, CERM, CERI, Anxiety state, Bruxism Index, and Self‐reported Bruxism Scale)

	1	2	3	4	5	6	7	8	9
**∆** Physical activity		0.129	−0.013	−0.47	0.004	0.033	−0.013	−0.105	−0.110
**∆** Social activity			0.205^a^	−0.097	0.067	0.155^b^	0.123	0.121	−0.043
**∆** Use of daytime social networks				0.102	0.403^a^	0.011	0.635^a^	0.559^a^	0.366^a^
**∆** Use of social networks at night					0.322^b^	0.067	0.108	0.246^a^	0.553^a^
**∆** CERM						0.182^a^	0.371^a^	0.384^a^	0.502^a^
**∆** CERI							−0.003	0.002	0.052
**∆** Anxiety state								0.477^a^	0.467^a^
**∆** Bruxism index									0.493^a^
**∆** Self‐reported Bruxism Scale									

Note

N = 213.

∆: change T0‐T1.

^a^ Correlation is significant at the 0.01 level.

^b^ Correlation is significant at the 0.05 level.

### 3.4 Predictors of increased bruxism

A hierarchical multiple regression was performed to determine whether the sum of increased social network use, increased mobile device use, and increased state anxiety improved the prediction of the increase in Self‐reported Bruxism Scale (see Table 3 for full details of each regression model). The complete model of increased social network use, increased mobile device use, and increased state anxiety (Model 3) was statistically significant, *R* ^2^ = 0.517, F(1209) = 74.5, *P* < .01; adjusted *R* ^2^ = 0.510. The addition of the variable of increased mobile device use to the prediction (Model 2) led to a statistically significant increase in R^2^ of 0.126, F(1, 210) = 40.161, *P* < .01. The addition of increased night‐time social network use to the prediction (Model 3) also led to a statistically significant increase in R^2^ of 0.174, F(1, 209) = 75 082, *P* < .01.

**TABLE 3:** Prediction of hierarchical multiple regression ∆ Self‐reported bruxism from ∆Anxiety state, ∆CERM, and ∆Use social networks night

Variable	∆ Self‐reported bruxism					
	Model 1		Model 2		Model 3	
	B	β	B	β	B	β
Constant	9.8**		9.4**		15.4**	
∆ Anxiety state	1**	0.46	0.69**	0.32	0.71**	0.33
∆ CERM			1.11**	0.38	0.69**	0.23
∆ Use social networks night					0.54**	0.44
R^2^	0.218		0.343		0.517	
F	58.753**		54.909**		74.547**	
∆R^2^	0.218		0.126		0.176	
∆F	58.753**		40.161**		75.082**	

Note

N = 213. ∆ = increase.

** *P* < .01.

### 3.5 Use of social networks during the night, anxiety, and bruxism

The moderating role of the increase in the use of social networks at night was evaluated between the increase in anxiety state and the increase in the scale of self‐reported bruxism (Table 4). The results indicated that the moderation model explained 48% of the variation of the increase in the scale of self‐perceived bruxism. The interaction between anxiety state and the use of nocturnal social networks significantly increased the coefficient of determination (F = 65.344; ∆R^2^ = 1.1; *P* ≤ .01). As for conditional effects, the impact of the increased use of nocturnal social networks on the scale of self‐reported bruxism was significant for the low use of nocturnal social networks (t = 3. 73; *P* ≤ .01; 95% CI = [0.29, 0.94]), for the medium use of social networks at night (t = 7.99; *P* ≤ .01; 95% CI = [0.65, 1.07]), and for the high use of social networks at night (t = 7.38; *P* ≤ .01; 95% CI = [0.81, 1.40]).

**TABLE 4:** Moderation effects of increased use of social networks at night moderating factor between increased state anxiety and increased Self‐reported Bruxism Scale

	Effect	SE	*t*	*P*	LLCI	ULCI
Model						
*R* ^2^ = 0.48; *F* = 65.34; *P* ≤ .01						
**Λ** Anxiety state	0.51	0.21	2.46	< .01	0.10	0.91
**Λ** Use of night networks	0.24	0.19	1.25	.21	‐0.14	0.62
**Λ** Anxiety state ^*^ **Λ** Use of night networks	0.03	0.01	2.13	.03	0.01	0.05
Conditional effects						
Low **Λ** Use of night networks	0.61	0.16	3.73	< .01	0.29	0.94
Medium **Λ** Use of night networks	0.86	0.11	7.99	< .01	0.65	1.07
High **Λ** Use of night networks	1.10	0.15	7.38	< .01	0.81	1.40

Note

Bootstrap samples = 10 000. *R* ^2^ = Coefficient of determination.

Abbreviation: LLCI, lower level of the 95% confidence interval; SE, standard error; ULCI, upper level of the 95% confidence interval.

∆: change T0‐T1.

* Interaction of variables.

## 4 DISCUSSION

The results provide empirical support for the objectives formulated in our study. They emphasize the possible relevance of the lockdown impact on the lifestyle of adolescents. Our findings indicate a significant decrease in social activity^20^ and a marked sedentarism. Similar findings have been reported in other studies,^1^ in which they reflected a poor social life, a high prevalence of physical inactivity during lockdown,^5^ and more screentime.^5^ Our findings add to this evidence, as participants reported a tendency to abuse social networks, cell phones, and the internet.

Previous literature shows that psychological reactions to past epidemics and pandemics depend on individual vulnerability, such as developmental age, having special needs, a pre‐existing mental health condition, or being economically disadvantaged.^21^ The strong impact COVID‐19 is, however, having on the population in terms of psychological problems has already been documented, as a high percentage of the population has reported moderate to severe anxiety.^22^ Recent research has warned that psychological factors associated with the pandemic may lead to a higher risk of bruxism,^23^ a fact that was noted in our study but associated with other variables involved in the pandemic.

In this context, the regression model explains how the increase in bruxism corresponds to a 17.6% abuse of social networks, to a 12.6% increase in the use of mobile devices, and to a 21.8% increase in anxiety state levels. These results are innovative and underline the importance of studying this model in an integrated way.

Until now, these variables have been studied in a fragmented rather than unified manner. The increase in the use of social networks and in the use of mobile devices^2^, ^3^, ^4^ was positively correlated with higher levels of anxiety as were high levels of anxiety associated with bruxism,^9^, ^10^ but the studies were separate.

The results of our research highlight the importance of the use of social networks at night as a predisposing factor for bruxism. This is in line with the data reported in previous research because the using of social networks prior to going to bed can delay the onset of sleep and diminish its quality as the disconnection required for sleep is complicated by the stimulation of connecting to social networks and the prolongation of the waking state. Social networks are prioritized over sleep.^24^

At the same time, the role of sleep disorders in the appearance of bruxism^25^ is a subject that has generated great interest due to the ongoing controversy that exists on the subject. In previous research, it has been found that most of the episodes of sleep bruxism are observed during light non‐REM sleep and tend to occur in relation to recurrent microactivation within the so‐called cyclic alternating pattern, which is repeated every 20‐60 seconds during non‐REM sleep.^26^ The use of devices by adolescents, before falling asleep, is associated with a more discontinuous sleep, characterized by a greater number of night‐time awakenings.^27^

The contributions of this study should be evaluated after taking into account its limitations as well. Firstly, we used a convenience sample, which came from a specific segment of the child population in the Community of Madrid, and this limits the possibilities of generalizing the results. Secondly, we have analysed the role played by a set of variables such as lack of physical activity, poor social life, abuse of social networks, cell phones, and internet, but these represent only some of the many factors that could be involved in the development of parafunctions and bruxism. A possible third limitation comes from the use of self‐report measures, which can be affected by memory biases and responses based on social desirability. Fourthly, in the diagnosis of bruxism, both self‐report and clinical assessment produce totally sensitive but insufficiently specific results compared to instrumental reference assessment, such as electromyographic records.^28^ Furthermore, in the clinical inspection, the presence of hypertrophy of the masticatory muscles was noted, as well as indentations in the tongue or lip and/or a dawn line on the inside of the cheek. These signs, however, can also be a consequence of functional promotor activity, such as swallowing.^29^

With regard to dental wear, it is considered a multifactorial condition, which leads to the loss of dental hard tissue. It can be divided into subtypes of mechanical wear (wear and abrasion) and chemical wear (erosion). Due to its multifactorial aetiology, tooth wear can manifest itself in many different representations and therefore can be difficult to diagnose.^30^ For this reason, it is plausible that the record at this level may have been biased.

This research may have implications for clinical practice, such as the field of prevention in adolescent oral health, which requires an extra effort from dentists in times of pandemic to investigate the possible aetiologies of patients with parafunctions and bruxism. In the event that professionals detect screen abuse, it may be appropriate for them to inform families that they should monitor their child's time spent on smartphones and social networks, as this may have detrimental effects on general and oral health, specifically on anxiety state levels, physical inactivity, and the development or aggravation of parafunctions and bruxism. It is well known that a sedentary lifestyle is a risk factor for chronic diseases. On the other hand, bruxism could be a risk factor for negative consequences for oral health^28^ such as painful temporomandibular disorders (TMD), mechanical wear of teeth, prosthetic complications, and others.^25^ As with onychophagy, nail biting is a habit that can also be harmful to health, with effects such as loss of dental tissue and increased risk of infection by the spread of pathogens from the nails to the mouth. And finally, there is no lack of scientific evidence to support how anxiety can affect both psychological and systemic health.^31^

Parents should encourage more non‐device‐related activities and games. In addition, they have an excellent opportunity to interact positively with their children through physical closeness and to strengthen bonds that will be more beneficial than mobile and social network abuse. In order to do this, parents, however, must set an example with the use of these devices.

Future lines of research are required to verify the results in representative populations and to consider the possibility of implementing interventions among the adolescent population in the area of social network and smartphone abuse, in order to minimize its possible effects on systemic and oral health, as well as ascertaining whether these are lasting effects. There has been no lockdown in Spain as strict as in the early stages of 2020, but there are, however, still many restrictions that affect the social life of adolescents and their lifestyle in general.

## CONFLICTS OF INTEREST

The authors declare no conflict of interest.

## AUTHOR CONTRIBUTIONS

CARRILLO‐DIAZ, M contributed to conception and design and critically revised manuscript. ORTEGA‐MARTINEZ AR contributed to acquisition, drafted the manuscript, and critically revised the manuscript. ROMERO, M contributed to conception and design and drafted the manuscript. GONZALEZ‐OLMO, MJ contributed to conception and design, contributed to analysis and interpretation, drafted the manuscript, and critically revised the manuscript.

All authors gave their final approval and agree to be accountable for all aspects of the work.

## ETHICAL APPROVAL

All procedures performed in studies involving human participants were in accordance with the ethical standards of the institutional and/or national research committee and with the 1964 Helsinki Declaration and its later amendments or comparable ethical standards. This research is supported by Rey Juan Carlos University Ethics and Research Committee. Informed consent was obtained from all individual participants included in the study.

## Data Availability

All of the material is owned by the authors and/or no permissions are required. The datasets generated during and analysed during this study are not publicly available due to [national data protection law] but are available from the corresponding author on reasonable request.
